# Editorial: Childhood traumatic experiences: new clinical perspectives and interventions

**DOI:** 10.3389/fpsyg.2025.1570098

**Published:** 2025-06-17

**Authors:** Valeria Saladino, Vincenzo Auriemma, Valeria Verrastro, Goran Arbanas, Stefano Eleuteri

**Affiliations:** ^1^Department of Health Sciences, “Magna Graecia” University of Catanzaro, Catanzaro, Italy; ^2^Department of Political and Social Studies, University of Salerno, Fisciano, Italy; ^3^University Psychiatric Hospital Vrapče, Zagreb, Croatia; ^4^Department of Human and Social Sciences, Mercatorum University, Rome, Italy

**Keywords:** trauma, child abuse, neglect, maltreatment, parental relationship, interventions

An overwhelming number of children worldwide are exposed to traumatic experiences, such as psychological, physical and verbal abuse, as well as parental neglect, which impact their psychosocial functioning and development. The parent-child relationship is highly impactful for the holistic development of children and youths. For this reason, adverse, neglectful, and traumatic exposures might impact individuals' emotional wellbeing, quality of adult relationships and growth. Indeed, neglect is characterized as a kind of emotional, educational, and physical abuse, due to its long-term impact. Also, those who have experienced multiple forms of abuse over an extended period may suffer from a greater number and more severe set of symptoms associated with the trauma.

Abused children carry some of such experiences throughout their adolescence, a multifaceted phase of individuals, characterized from physical, psychological, and social development. Relational problems, behavioral difficulties and psychological vulnerabilities often stem from early traumatic experience roughens the process of developing one's sense of self as well as resilience.

Multiple childhood traumatic experiences are closely related to parent-child relations and are likely to be highly amenable to interventions in parent-child contexts. In the past few years research has greatly contributed to dealing with this Research Topic, giving important pointers for further considerations to researchers. Parental separation or abandonment can undoubtedly be a traumatic experience for a child, leading to significant repercussions. A study conducted in Tehran highlighted that orphaned adolescents exhibited greater reward sensitivity, higher impulsivity, emotional regulation difficulties, and attention deficits compared to their peers with parents. These challenges are attributed to the lack of affectionate care during childhood, underscoring the essential role of parental figures in emotional and cognitive development (Sadeghzadeh and Bagheri).

On the other hand, as suggested by Springer and Lueger-Schuster, also the parental drug use and caregiver attitudes influence the mentalization processes of children in foster care. The authors identify factors that support resilience and effective mentalization, helping to inform interventions that improve outcomes for children in foster care (Springer and Lueger-Schuster). Their longitudinal study investigates the psychological and emotional development of these children. Trauma represents a powerful experience that causes physical and mental harm to individuals. In this regard, a study on adolescents with hearing impairments (DHH) in Saudi Arabia revealed that 97% of these students had experienced at least one form of abuse or neglect. A strong correlation has emerged between maltreatment and psychological disorders such as depression and anxiety. Major risk factors for maltreatment included being male, critical socioeconomic conditions, and limited communication between hearing parents and DHH children. In this regard, the study suggests targeted interventions, such as educational programs on sign language, awareness campaigns, and integrated support between schools and mental health services, emphasizing the need for further longitudinal research (Hammad et al.).

Adverse childhood experiences (ACEs) significantly influence substance use, with low self-control acting as a key mediator. One of the studies, conducted among Ugandan adolescents, emphasizes the need for targeted interventions on self-control and the importance of individualized support for vulnerable youth (Namusoke et al.).

A part of the studies in the topic concentrate on the possible effectiveness of different experiences in decreasing problematic behavior in different children. In Italy, for example, a study explored the effectiveness of the e-Connect program, a digital version of the Connect Parent Group (CPG^®^), implemented with adoptive and foster parents of adolescents. The program, rooted in attachment and trauma-based principles, demonstrated a reduction in parental stress and adolescents' emotional and behavioral difficulties, while improving the understanding of problematic behaviors and strengthening the parent-child relationship. While the results appear promising, notable limitations, such as a small sample size and the lack of in-depth statistical analysis, emerged. Nevertheless, the study highlights the significant potential of the program as an effective and accessible tool for vulnerable families (Pace et al.). Trauma-informed approaches are essential for supporting vulnerable populations in both educational and family contexts. In a specific study, the authors have found that for refugee students, trauma-sensitive school concepts create safe environments that address psychological wellbeing and academic success by fostering resilience and understanding among educators and policymakers (Lembke et al.). Similarly, another research concentrated on understanding how tailored parenting interventions for African American families impacted by IPV can highlight the need for culturally informed strategies. These include strengths-based approaches, racial socialization, peer support networks, and access to social services, all aimed at empowering mothers and fostering long-term support systems (Cervantes et al.). Both frameworks emphasize the role of systemic, empathetic solutions in addressing trauma and promoting equity.

Over the years, interest in this topic has grown significantly, pushing researchers to deepen the validity of questionnaires aimed at assessing adolescent wellbeing. For instance, Olàh et al. proposed a study to examine the validity of the ACE-10 questionnaire among vulnerable adolescents in the child welfare system in Hungary, comparing it with a modified 9-item version (ACE-9). The latter proved to be more suited to the studied population, showing greater internal consistency and strong correlations with psychological variables such as emotional and behavioral difficulties. The research emphasizes the need for developing dependable diagnostic tools in order to be able to properly recognize traumatic events and be able to plan adequate interventions.

Nevertheless, one must not underestimate the difficulties that parents encounter when they seek to deal with the inappropriate behaviors displayed by their children. A critical topic is understanding and managing children's behavior by their parents. In this regard, an integrated approach to Parent-Child Interaction Therapy (PCIT), combined with the attachment-based Circle of Security-Parenting (COS-P) program, has been developed to address problematic behaviors in maltreated children. This model enhances parents' emotional understanding and reduces children's problematic behaviors, increasing family participation. However, its implementation requires thorough therapist training and further study to evaluate its long-term effectiveness (Belanger et al.). A different approach is proposed by a study from Montenegro et al., which explores the impact of trauma-focused group therapy incorporating Karate-Do on children and adolescents affected by war trauma. It presents a case study demonstrating how this therapy promotes emotional resilience, self-regulation, and social integration. Using martial arts as a therapeutic tool, the program combines physical activity with psychological support to address trauma symptoms. Results suggest positive outcomes, including improved mental wellbeing and coping mechanisms.

Understanding and addressing childhood traumatic experiences are not merely a matter of individual support but a social commitment. It requires an integrated approach involving families, professionals, and institutions. Only through such efforts can the wellbeing of future generations be improved, fostering healthier, and more constructive parental relationships.

